# Role of Cystathionine Gamma-Lyase in Immediate Renal Impairment and Inflammatory Response in Acute Ischemic Kidney Injury

**DOI:** 10.1038/srep27517

**Published:** 2016-06-08

**Authors:** Lajos Markó, István A. Szijártó, Milos R. Filipovic, Mario Kaßmann, András Balogh, Joon-Keun Park, Lukasz Przybyl, Gabriele N’diaye, Stephanie Krämer, Juliane Anders, Isao Ishii, Dominik N. Müller, Maik Gollasch

**Affiliations:** 1 Experimental and Clinical Research Center, a joint cooperation between the Charité Medical Faculty and the Max-Delbrück Center (MDC) for Molecular Medicine, Berlin, Germany; 2 Max-Delbrück Center (MDC) for Molecular Medicine, Berlin, Germany; 3 Friedrich-Alexander-University of Erlangen-Nürnberg, Department of Chemistry and Pharmacy, Erlangen, Germany; 4 Hannover Medical School, Hannover, Germany; 5 German Institute of Human Nutrition, Potsdam-Rehbrücke, Germany; 6 Department of Biochemistry, Graduate School of Pharmaceutical Sciences, Keio University, Tokyo, Japan; 7 Charité Campus Virchow, Nephrology/Intensive Care, Berlin, Germany

## Abstract

Hydrogen sulfide (H_2_S) is known to act protectively during renal ischemia/reperfusion injury (IRI). However, the role of the endogenous H_2_S in acute kidney injury (AKI) is largely unclear. Here, we analyzed the role of cystathionine gamma-lyase (CTH) in acute renal IRI using CTH-deficient (*Cth*^−/−^) mice whose renal H_2_S levels were approximately 50% of control (wild-type) mice. Although levels of serum creatinine and renal expression of AKI marker proteins were equivalent between *Cth*^−/−^ and control mice, histological analysis revealed that IRI caused less renal tubular damage in *Cth*^−/−^ mice. Flow cytometric analysis revealed that renal population of infiltrated granulocytes/macrophages was equivalent in these mice. However, renal expression levels of certain inflammatory cytokines/adhesion molecules believed to play a role in IRI were found to be lower after IRI only in *Cth*^−/−^ mice. Our results indicate that the systemic CTH loss does not deteriorate but rather ameliorates the immediate AKI outcome probably due to reduced inflammatory responses in the kidney. The renal expression of CTH and other H_2_S-producing enzymes was markedly suppressed after IRI, which could be an integrated adaptive response for renal cell protection.

Hydrogen sulfide (H_2_S) has been recognized as a toxic gas for many years until Warenycia *et al*. discovered the endogenous production of H_2_S in the rat brain^1^. Endogenous H_2_S is mainly produced by enzymes of the transsulfuration pathway, cystathionine gamma-lyase (CTH or CSE) and cystathionine beta-synthase (CBS). The third enzyme, 3-mercaptopyruvate sulfurtransferase (MPST or MST), can also contribute to endogenous H_2_S production in the presence of reductants using 3-mercaptopyruvate as a substrate^2^,^3^,^4^. Although the physiological role of MPST in mammalian tissues is less well characterized, MPST might contribute to H_2_S production in the brain or modulation of cardiovascular functions^5^,^6^.

Several studies demonstrated that H_2_S could exert protective effects in the cardiovascular system. In particular, H_2_S has emerged as potential therapeutics^3^ for ischemia/reperfusion injury (IRI) of different organs^2^. This knowledge mainly relies on the results from animal studies using H_2_S/H_2_S donor molecules or pharmacological inhibitors of H_2_S-producing enzymes^7^. However, the physiological levels of H_2_S in tissues have been a matter of debate because of methodological difficulties in measuring accurate, reliable, and reproducible H_2_S levels in biological samples. In addition, pharmacological CTH/CBS inhibitors have been used at suspicious high concentrations *in vivo* and *in vitro*^8^, which might cause the lack of specificity in enzyme inhibition^2^,^9^. Therefore, transgenic animals have been recently developed to elucidate the role of H_2_S in health and disease^10^,^11^.

CTH is highly expressed in the kidney and several studies demonstrated that H_2_S could exert protective effects in renal IRI. Han *et al*. found that NaHS administration to IRI mice accelerated the recovery from renal dysfunction and impaired tubular morphology, whereas the administration of dl-propargylglycine (PAG), an inhibitor of CTH, delayed it^12^. NaHS administration had beneficial effects on renal IRI^13^ and gentamicin-induced acute kidney injury (AKI) in rats^14^. Tan *et al*. suggested that the beneficial effects of endogenously produced H_2_S in AKI are, at least in part, mediated by toll-like receptors (TLRs)-mediated inflammatory response and apoptosis^15^. Chen *et al*. showed that exogenous H_2_S reduces kidney injury from urinary-derived sepsis in rabbits, which is associated with decreased TNF-α expression in the kidneys^16^. On a conceptual level, these results are consistent with recent findings by Bos *et al*. who reported that their CTH-deficient (*Cth*^−/−^) mice display aggravated renal IRI^10^,^17^ when our study was underway. The authors concluded that CTH protects against renal IRI, likely by modulating oxidative stress through the production of H_2_S. Autosomal-recessive cystathioninuria (OMIM 219500), which is considered as a benign biochemical anomaly, is caused by homozygous or compound heterozygous mutations in *CTH* (OMIM 607657) and has a relatively high prevalence (1 per 14,000 live births)^18^ though with somewhat lower incidence in other reports^19^,^20^. Therefore, both the findings of Bos *et al.* and ours could have important implications for humans.

Here, we used our *Cth*^−/−^ mice on a C57BL/6J background^11^ to elucidate the function of CTH in renal IRI. We found that the systemic CTH loss does not deteriorate the immediate outcome of AKI. Instead *Cth*^−/−^ mice displayed reduced renal damage and renal expression of inflammatory cytokines/adhesion molecules after IRI, compared to littermate control (wild-type; *Cth*^+/+^) and heterozygous (*Cth*^+/−^) mice.

## Results

### The mRNA Expression of H_2_S-Generating Enzymes in the Kidney

**Figure 1:**
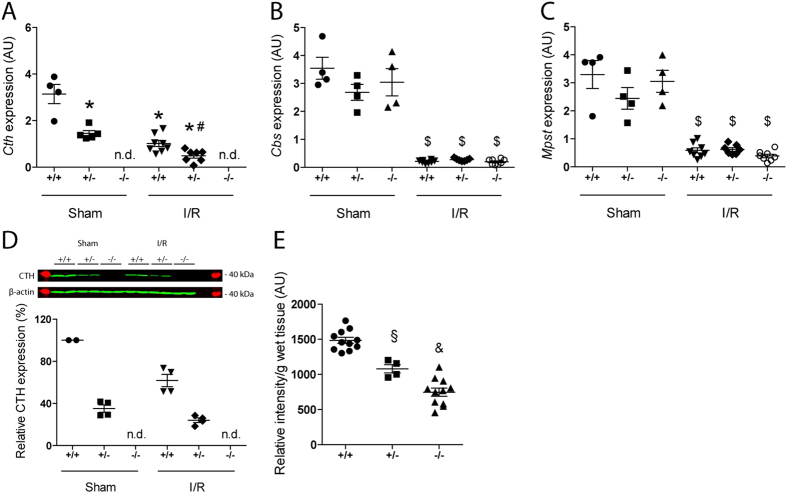
Renal expression of H_2_S-producing enzymes and H_2_S. Gene expression levels of (**A**) cystathionine gamma-lyase (*Cth*), (**B**) cystathionine beta-synthase (*Cbs*) and (**C**) 3-mercaptopyruvate sulfurtransferase (*Mpst*) in sham- and ischemia/reperfusion (I/R)-injured kidneys of wild-type (*Cth*^+/+^), heterozygous (*Cth*^+/−^) and CTH-deficient (*Cth*^−/−^) mice. Values plotted are mean ± SEM (*n* = 4 in sham-operated groups, *n* = 8 in I/R-injured groups). *P < 0.05 vs. sham-operated *Cth*^+/+^ and sham-operated Cth^+/−^; ^#^P < 0.05 vs. I/R-injured *Cth*^+/+^ and sham-operated *Cth*^+/−^; ^$^P < 0.05 vs. sham-operated *Cth*^+/+^, *Cth*^+/−^ and *Cth*^−/−^ mice. (**D**) Relative CTH protein levels of sham- and I/R-injured kidneys of *Cth*^+/+^, *Cth*^+/−^ and *Cth*^−/−^ mice. Values plotted are mean ± SEM (*n* = 4 each). Mean CTH density of two *Cth*^+/+^ kidneys on each gel was set to 100% and relative density was calculated for the rests. (**E**) Levels of H_2_S in intact kidneys of *Cth*^+/+^, *Cth*^+/−^ and *Cth*^−/−^ mice. Values plotted are mean ± SEM (*n* = 11 for *Cth*^+/+^ and *Cth*^−/−^, and *n* = 4 for *Cth*^+/−^). ^&^P < 0.01 vs. *Cth*^+/+^ and *Cth*^+/−^; ^§^P < 0.05 vs. *Cth*^+/+^ and *Cth*^−/−^. AU, arbitrary units. n.d., not detectable.

Levels of renal *Cth* mRNA in heterozygous (*Cth*^+/−^) mice were approximately half of those in *Cth*^+/+^ mice (Fig. 1A). In both *Cth*^+/+^ and *Cth*^+/−^ mice subjected to IRI, renal *Cth* mRNA levels declined to one third of their initial expression levels (Fig. 1A). No *Cth* mRNA was detectable in the kidneys of *Cth*^−/−^ mice (Fig. 1A). Renal mRNA levels of two other H_2_S-producing enzymes, CBS and MPST, were not different among all three *Cth* genotypes. These levels declined markedly after IRI and the levels after IRI were also not different among *Cth* genotypes (Fig. 1B,C).

### Protein Expression of CTH and Endogenous H_2_S Levels in the Kidney

Levels of renal CTH protein in heterozygous (*Cth*^+/−^) mice were ~40% of those in *Cth*^+/+^ mice (Fig. 1D) as previously reported^11^. Similar to its mRNA level changes, renal CTH protein levels declined after IRI in both *Cth*^+/+^ and *Cth*^+/−^ mice and no CTH protein was detectable in the kidneys of *Cth*^−/−^ mice (Fig. 1D). Next, we measured endogenous H_2_S levels in the kidney to assess the impact of systemic CTH deletion on H_2_S production. Kidneys of *Cth*^+/−^ and *Cth*^−/−^ mice displayed approximately 30% and 50% reduced H_2_S levels, respectively, compared to those of *Cth*^+/+^ mice (Fig. 1E).

### The impact of CTH loss in Renal Damage after IRI

**Figure 2:**
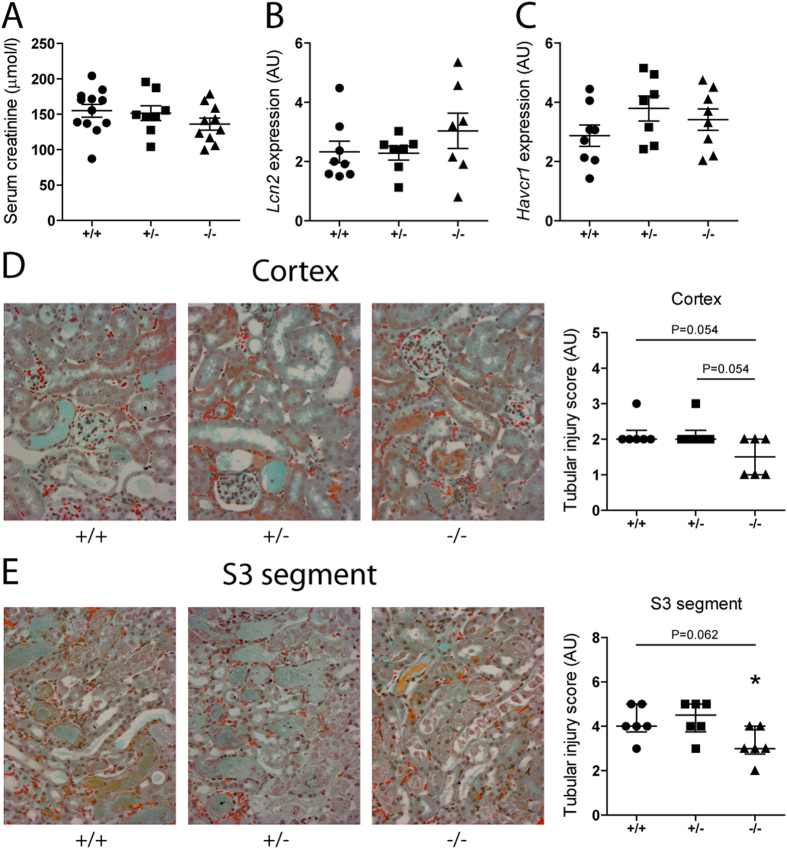
Renal damage after ischemia/reperfusion (I/R) injury. (**A**) Serum creatinine levels (*n* = 12, 8, and 10 for *Cth*^+/+^, *Cth*^+/−^, and *Cth*^−/−^ mice, respectively) and mRNA levels of (**B**) kidney injury marker lipocalin 2 (*Lcn2*) and (**C**) hepatitis A virus cellular receptor 1 (*Havcr1*) in I/R-injured kidneys of *Cth*^+/+^, *Cth*^+/−^, and *Cth*^−/−^ mice (n = 8, 7, and 8, respectively). Values plotted are mean ± SEM. (**D**) Representative cortical images of Masson’s trichrome stain on sections of I/R-injured kidneys of *Cth*^+/+^, *Cth*^+/−^, and *Cth*^−/−^ mice (×200). Right hand side is semi-quantification of cortical tubular injury. Values plotted are median ± interquartile range (*n* = 6 each). (**E**) Representative S3 segment images of Masson’s trichrome stain on sections of I/R-injured kidneys of *Cth*^+/+^, *Cth*^+/−^, and *Cth*^−/−^ mice (×200). Right hand side is semi-quantification of tubular injury. Values plotted are median ± interquartile range (*n* = 6 each). *P < 0.05 vs. *Cth*^+/−^ mice. Red dots are red blood cells. AU, arbitrary units.

To get insights into (patho)physiological roles of CTH in renal IRI, we performed comparative *in vivo* studies using *Cth*^+/+^, *Cth*^+/−^ and *Cth*^−/−^ mice. Twenty-four hours after ischemia, *Cth*^−/−^ mice showed somewhat lower serum creatinine levels (136 μmol/l average) compared to *Cth*^+/−^ mice (152 μmol/l average) and *Cth*^+/+^ mice (155 μmol/l average) although the differences were not statistically significant (overall ANOVA P = 0.30; Fig. 2A). Renal mRNA levels of two sensitive AKI markers, lipocalin 2 (*Lcn2*; also known as neutrophil gelatinase-associated lipocalin [*Ngal*]) and hepatitis A virus cellular receptor 1 (*Havcr1*; also known as kidney injury molecule 1 [*Kim1*]), were not different between *Cth*^+/+^, *Cth*^+/−^, and *Cth*^−/−^ mice (Fig. 2B,C). To assess the degree of tubular damage after ischemic AKI, kidney sections were stained and examined by an experienced renal pathologist who was unaware of *Cth* genotypes. Histological analyses and semi-quantitative scoring revealed a moderate amelioration in cortical tubular damage after renal IRI in *Cth*^−/−^ mice compared to *Cth*^+/−^ or *Cth*^+/+^ mice (Fig. 2D). Histological analysis of the S3 segments of the proximal tubules in the outer stripe of outer medulla, especially vulnerable loci against renal IRI, identified that *Cth*^−/−^ mice exhibit less tubular necrosis and less occlusions of tubular lumen with cellular debris, compared to *Cth*^+/−^ or *Cth*^+/+^ mice (Fig. 2E). These parameters were comparable among sham-operated mice with all *Cth* genotypes (Supplementary Figure 1A–C). It is notable that all *Cth*^+/+^, *Cth*^+/−^ and *Cth*^−/−^ mice that underwent surgery survived in this study.

### The impact of CTH loss in Cellular Infiltration to the Kidneys after IRI

**Figure 3:**
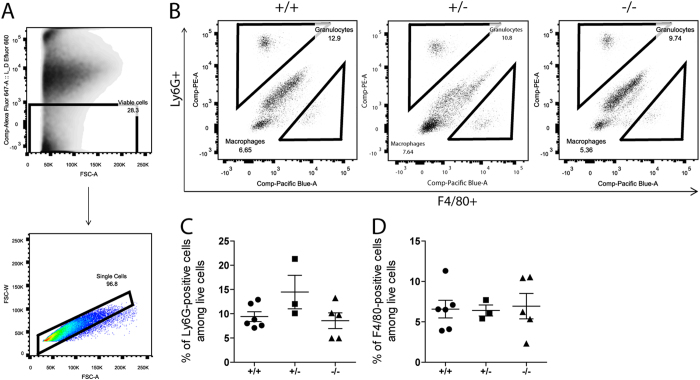
Flow cytometric analysis of renal granulocyte and macrophage infiltration. (**A**) Pre-gating on live cells using Fixable Viability Dye eFluor 660 and further gating on single cells. (**B**) Representative flow cytometry data of infiltrating Ly6G-positive cells (granulocytes) and F4/80-positive cells (macrophages) in I/R-injured kidneys of *Cth*^+/+^, *Cth*^+/−^, and *Cth*^−/−^ mice. Quantification of infiltrating (**C**) Ly6G-positive cells and (**D**) F4/80-positive cells. Values plotted are mean ± SEM (*n* = 6, 3 and 5 for *Cth*^+/+^, *Cth*^+/−^, and *Cth*^−/−^ mice, respectively).

Renal IRI is known to associate with infiltration of granulocytes, monocytes/macrophages and other immune cells immediately after reperfusion, which contributes to inflammation and subsequent repair in injured kidneys^21^. Therefore, we characterized granulocytes and macrophages in renal IRI by flow cytometry. Whole kidney cell suspensions were immunolabelled for Ly6G and F4/80 as markers for granulocytes and macrophages, respectively. Among pre-gated singlet live cells (Fig. 3A), Ly6G-positive & F4/80-negative granulocytes as well as Ly6G-negative & F4/80-positive macrophages were detected (Fig. 3B). There were no significant differences in both granulocyte and macrophage populations between *Cth*^+/+^, *Cth*^+/−^, and *Cth*^−/−^ kidneys at 24 h after IRI (Fig. 3C and D) or after sham surgery (Supplementary Figure 2A–C). We next performed immunohistochemistry to detect IRI-induced granulocyte infiltration (Supplementary Figure 3A)^22^. In the outer medulla after IRI, average Ly6B-positive cell numbers per view field were 12 in *Cth*^−/−^ mice while 13 and 19 in *Cth*^+/−^ and *Cth*^+/+^ mice, respectively, although the differences were not statistically significant (P = 0.501, Supplementary Figure 3B). Furthermore, renal levels of *S100a8/a9* mRNAs for calprotectin, a heterodimeric protein that was recently found to co-localize with Ly6G in granulocytes after AKI and playing a crucial part in controlling M2 macrophage-mediated renal repair following IRI^23^, were also not significantly different (Supplementary Figure 3C,D).

### The impact of CTH loss in Expression of Cytokines, Chemokines, and Adhesion Molecules

**Figure 4:**
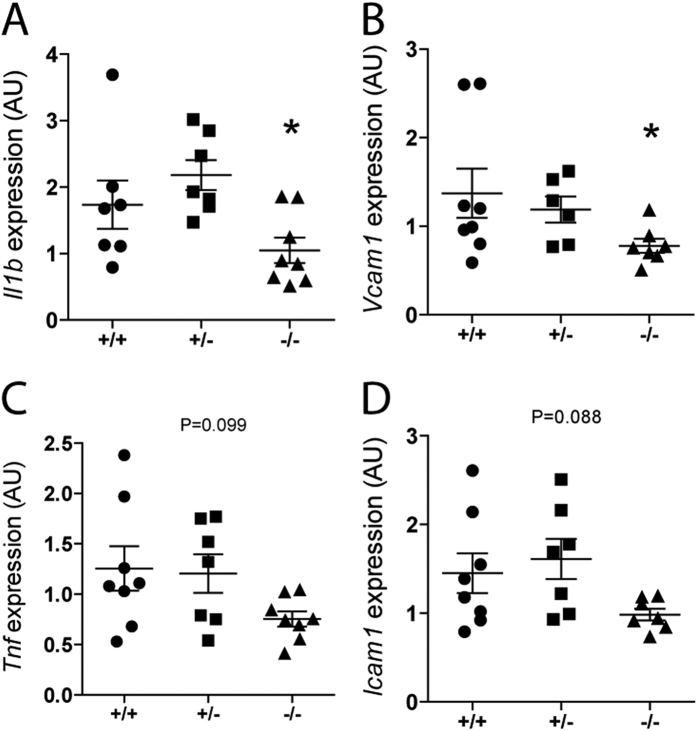
Renal gene expression of cytokines and adhesion molecules. Gene expression levels of (**A**) interleukin (*Il*))1-beta, (**B**) vascular cell adhesion molecule (*Vcam*)1, (**C**) tumor necrosis factor alpha (*Tnf*) and (**D**) intercellular adhesion molecule (*Icam*)1 in ischemia/reperfusion-injured kidneys of *Cth*^+/+^, *Cth*^+/−^, and *Cth*^−/−^ mice. Values plotted are mean ± SEM (n = 8, 7, and 8 for *Cth*^+/+^, *Cth*^+/−^, and *Cth*^−/−^ mice, respectively). *P < 0.05 vs. *Cth*^+/−^ mice. AU, arbitrary units.

Production of inflammatory molecules is maintained low in the normal kidney but is markedly increased under pathophysiological conditions such as ischemia^21^. We measured mRNA levels of several molecules involved in long-term outcome/repair after renal IRI. Renal expression of interleukin 1-beta (*Il1b*) and vascular cell adhesion molecule 1 (*Vcam1*) after IRI was significantly lower in *Cth*^−/−^ mice compared to *Cth*^+/−^ mice (Fig. 4A,B). Also, renal expression of tumor necrosis factor-alpha (*Tnf*) and vascular cell adhesion molecule 1 (*Vcam1*) was similarly lower in *Cth*^−/−^ mice compared to *Cth*^+/−^ mice (overall ANOVA P = 0.099 and P = 0.088, respectively) (Fig. 4C,D). Renal expression of other important cytokines/chemokines such as interleukin 6 (*Il6),* chemokine (C-X-C motif) ligand 2 (*Cxcl2*), and chemokine (C-C motif) ligand 2 (*Ccl2*), were not altered among *Cth* genotypes (Supplementary Figure 4A–C).

### The impact of CTH loss in *In Vitro* Macrophage Polarization

**Figure 5:**
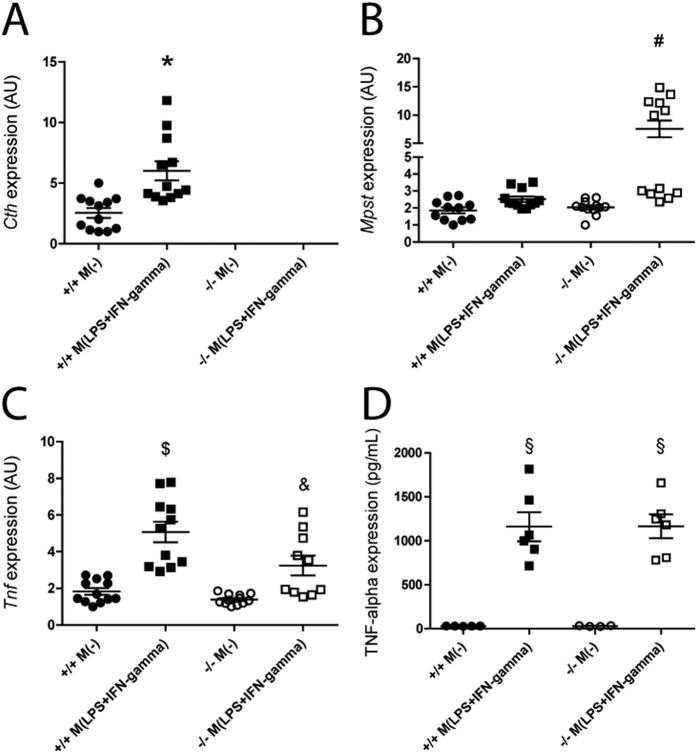
Gene expression of hydrogen sulfid producing enzymes and tumor necrosis alpha (Tnf) in bone marrow (BM)-derived macrophages. Gene expression levels of (**A**) cystathionine gamma-lyase (*Cth*), (**B**) 3-mercaptopyruvate sulfurtransferase (*Mpst*) and (**C**) *Tnf* in non-polarized M(−) and lipopolysaccharide (LPS) and interferon (IFN)-gamma polarized *Cth*^+/+^ and *Cth*^−/−^ BM-derived macrophages. Values plotted are mean ± SEM (*n* = 12 of each group, 2 independent measurement, 6 biological repetition/experiment). *P < 0.05 vs. *Cth*^+/+^ M(−). ^#^P < 0.05 vs. *Cth*^+/+^ M(−), *Cth*^+/+^ M(LPS+IFN-gamma) and *Cth*^−/−^ M(−). ^$^P < 0.05 vs. *Cth*^+/+^ M(−), *Cth*^−/−^ M(−) and *Cth*^−/−^ M(LPS+IFN-gamma), ^&^P < 0.05 vs. *Cth*^+/+^ M(−), *Cth*^+/+^ M(LPS+IFN-gamma) and *Cth*^−/−^ M(−). AU, arbitrary units. (**D**) TNF-alpha concentration in the supernatant of the cultured BM-derived macrophages. Values plotted are mean ± SEM (n = 5 and 6 for non-polarized and polarized macrophage group, respectively). ^§^P < 0.05 vs. *Cth*^+/+^ M(−) and *Cth*^−/−^ M(−).

Although the proportion of infiltrating macrophages after IRI was not significantly different (Fig. 3D), renal mRNA expression of IL1-beta and TNF-alpha, the two major inflammatory cytokines of macrophage origin, was lower or in *Cth*^−/−^ mice (Fig. 4A,C). We hypothesized that macrophage polarization is disturbed by the lack of CTH, and thus investigated *Tnf* induction by the lipopolysaccharide (LPS)/interferon (IFN)-gamma *in vitro* treatment of bone marrow (BM)-derived macrophages from *Cth*^+/+^ and *Cth*^−/−^ mice. *Cth* expression was induced while *Mpst* expression was not altered by LPS/IFN-gamma treatment in BM-derived macrophages from *Cth*^+/+^ mice (Fig. 5A,B). In contrast, *Mpst* expression was significantly induced by the same treatment in macrophages from *Cth*^−/−^ mice (Fig. 5B), and *Cbs* expression was not detectable in macrophages from either mice (data not shown). Under such conditions, *Tnf* expression was markedly induced by LPS/IFN-gamma treatment of both *Cth*^+/+^ and *Cth*^−/−^ macrophages, and the levels were significantly lower in *Cth*^−/−^ macrophages (Fig. 5C), although the supernatant TNF-alpha concentrations of activated macrophages were comparable between *Cth*^+/+^ and *Cth*^−/−^ mice (Fig. 5D).

## Discussion

A number of studies have demonstrated the cytoprotective effects of H_2_S in myocardial, liver, brain, pulmonary, and renal IRI (reviewed by Nicholson and Calvert)^2^. Most of these studies utilized Na_2_S/NaHS as exogenous H_2_S donors and PAG as a non-specific CTH inhibitor. To overcome pharmacokinetic problems in H_2_S donor applications and specificity issues of PAG, two research groups have independently generated mice in which *Cth* genes have been differentially deleted^10^,^11^. In our study, we investigated the pathophysiological roles of CTH in renal IRI using one of those *Cth*^−/−^ mice and their littermate *Cth*^+/−^ and *Cth*^+/+^ mice as controls; all were the offspring from the mating between *Cth*^+/−^ males and *Cth*^+/−^ females that had been backcrossed over 10 generations onto a C57BL/6 background^11^. We found that the lack of CTH does not cause aggravated immediate renal functional impairments after IRI as assessed by serum creatinine levels (Fig. 2A) and renal expression of sensitive AKI markers, Lcn2 and Havcr1 (Fig. 2B,C). Our histological examinations rather identified a moderate amelioration in renal tubular damage in *Cth*^−/−^ mice (Fig. 2D,E).

While our study was underway, Bos *et al.* published findings with their *Cth*^−/−^ mice (on a mixed strain background; the sex of mice used is not indicated) investigating the role of CTH-derived H_2_S in renal IRI^17^. They found that CTH deficiency aggravated kidney damage after IRI, which was associated with increased mortality^17^ however, we did not observe such severe systemic damage after renal IRI. The reasons for this discrepancy are possibly multifaceted. *First*, their *Cth*^−/−^ mice display age-dependent hypertension (15–20 mmHg higher systolic blood pressure *vs Cth*^+/+^ mice only after 7 weeks of age) and sex-related hyperhomocysteinemia in which females have six times the plasma homocysteine levels (120 *vs* 20 μM) in males^10^,^17^, both of which are caused by unknown mechanisms. Hypertension *per se* has deleterious effects on renal IRI^24^,^25^ nevertheless, hypertension was not properly treated in their studies^17^. This affair makes it difficult to assess the impact of reduced renal H_2_S production over elevated blood pressure on the outcome of IRI; fortunately, our *Cth*^−/−^ mice display systolic normotension^11^. This fact may, at least in part, underlie differences between their and our findings. It should be noted that our *Cth*^−/−^ males and females display similar serum levels of homocysteine (104–151 μM)^11^ the reasons for this difference are yet unknown but may depend on differences in genetic backgrounds and/or nutritional conditions. *Second*, Bos *et al.* performed renal ischemia by clamping both (right and left) renal arteries for 30 min, whereas we performed uninephrectomy by clamping the renal artery of the remnant left kidney for 20 min^17^. Despite the differences in surgical protocols, serum creatinine levels at 24 h after IRI were equivalent. But, importantly, all mice that underwent surgery survived in our study while Bos *et al.* observed 35% mortality only in *Cth*^−/−^ mice^17^. *Third*, we used a temperature controller with heating pads to maintain a stable core temperature (which was measured continuously during surgery by a rectal probe) whereas Bos *et al.* used only heating pads and lamps^17^. It is well known that fluctuations in core body temperature contribute to variability in IRI and the way of maintaining body temperature during ischemia has a major impact on the outcome of IRI^26^. *Fourth*, their *Cth*^−/−^ mice showed a massive (91%) reduction in renal H_2_S production compared to *Cth*^+/+^ mice^17^ while our *Cth*^−/−^ mice showed only 50% reduction (Fig. 1E). Although the methods used for H_2_S measurement substantially differ between the two studies and this precludes the direct comparison, >90% reduction is surprising *per se*, considering the facts that (i) *Cth*^−/−^ kidney still expresses CBS and MPST, (ii) (increased/activated) CBS could compensate for H_2_S production when CTH is inhibited or abrogated (though we did not observe compensatory *Cbs* mRNA induction; (Fig. 1B) and (iii) renal *Cbs*/*Mpst* expression was markedly down-regulated by ischemia/reperfusion irrespective of *Cth* genotypes (Fig. 1B,C)^11^,^12^,^17^,^27^,^28^. A previous study mentioned that the reduction in CBS (rather than CTH) activity may serve as the major contributor for endogenous H_2_S level reduction during renal IRI^29^.

Despite such differences, we also found some agreement with previous studies by Bos *et al.*^17^ and others^27^,^30^. First, renal expression (either gene or protein) of both CTH and CBS were suppressed after renal IRI (Fig. 1A,B). It might be noteworthy that the partial or complete loss of CTH did not cause compensatory induction (or reduced repression of expression) of CBS (or MPST) during IRI (Fig. 1B,C) at least on mRNA level. Second, both Bos *et al.* and we did not find significant differences between *Cth* genotypes in the numbers of granulocyte infiltrated into injured kidneys of IRI mice (Fig. 3C and Supplementary Figure 3A–C)^17^. We also counted the numbers of F4/80-positive macrophages infiltrated into injured kidneys of IRI mice and found that macrophages behave similar to granulocytes (Fig. 3D). In contrast, renal expression of *Tnf*, *Il1b*, *Icam1,* and *Vcam1* after IRI were lower (though overall ANOVA was just P = 0.099 and 0.088 for *Tnf* and *Icam1*, respectively) in *Cth*^−/−^ mice compared to *Cth*^+/−^ mice (Fig. 4A–D). TNF-alpha was initially discovered as a LPS-induced macrophage product^31^. It is also released during IRI and acts as a potent pro-inflammatory cytokine^32^, and in line, the blockade of TNF-alpha signaling is a novel promising therapeutic target in renal IRI^33^. Although intrinsic renal cells also secret TNF-alpha upon injury, monocytes/classically activated macrophages are considered as the main source of TNF-alpha in early renal IRI^34^. We found that CTH deficiency alters *Tnf* expression in LPS/IFN-gamma-stimulated BM-derived macrophages that intrinsically differ from LPS-stimulated peritoneal macrophages^10^,^35^. However, the supernatant TNF-alpha concentrations did not differ between both groups, which questions the physiological relevance of this finding. Meanwhile, renal expression of other cytokines that are known to play a role in renal IRI^36^ (Il6, Cxcl2, and Ccl2) were not distinguishable between *Cth* genotypes (Supplementary Figure 4A–C).

Our findings are in contrast to previous results by others who use PAG for CTH inhibition. Tripatara *et al.* found that single intraperitoneal administration of PAG (50 mg/kg, 1 h before ischemia) prevented the renal recovery from IRI (45-min ischemia/72-h reperfusion) in a rat bilateral ischemia model^37^. More recently Han *et al.* showed similar deteriorative effects of PAG in renal IRI (50 mg/kg daily (i.p.), beginning 2 days after ischemia) in mice^12^. However, PAG (5 mg/kg (i.p.), twice a day for 4 successive days) exhibited nephroprotective effects in the cisplatin model of AKI in rats^38^. Similar protective effects of PAG (50 mg/kg (i.p.) at 2 h after adriamycin injection) have been observed in adriamycin-induced nephrotoxicity in rats^39^. Whereas these kidney injury models differ, they point out that PAG treatment can have multiple effects depending on the renal injury models. Moreover, the specificity of this widely used CTH inhibitor and relatively late time points after reperfusion are a matter of concern. Our model is of particular interest because we used a genetic approach to abrogate CTH specifically and investigated acute renal post-ischemic injury after 24 h, a time point where serum creatinine levels are the highest and renal *Cth*/*Cbs* expression levels are the lowest^17^.

Numerous studies have revealed cytoprotective/anti-oxidative/anti-inflammatory roles of H_2_S, but some studies also have identified pro-inflammatory roles of H_2_S that accelerate inflammatory responses; for example, Ang *et al.* previously reported that caerulein-induced acute pancreatic damage as well as its associated lung injury was ameliorated in *Cth*^−/−^ mice compared to *Cth*^+/+ ^40. It is possible that CTH-produced H_2_S may act as a pro-inflammatory factor in renal IRI. In addition, further studies should also clarify the impact of high levels of cystathionine and homocysteine and low levels of taurine that are common in *Cth*^−/−^ mice^11^ on the outcome of renal IRI^41^,^42^,^43^. In conclusion, the systemic loss of CTH in mice caused approximately 50% reduction in renal H_2_S levels but did not influence immediate outcomes of ischemic AKI; however, it reduced tubular damage moderately and suppressed the renal expression of inflammatory cytokines. Future studies should clarify the role of CTH on the long-term outcome of renal impairment in AKI.

## Methods

### Mice

*Cth*^+/−^ and *Cth*^−/−^ mice were generated and characterized earlier^11^. In this study, *Cth*^+/−^ males and females were bred to obtain *Cth*^+/+^, *Cth*^+/−^, and *Cth*^−/−^ littermates. Mice were allowed free access to standard chow and water. The mice were kept in a 12:12-h light-dark cycle. All works involving animals have been approved by the Berlin Animal Review Board in 2012 (No. G 0444/12) and conducted in accordance with the American Physiological Society standards.

### Renal IRI Model

Male mice (age between 12–15 weeks) were used. Anesthesia was performed with isoflurane (2.3%) in air (350 ml/min)^44^. Each mouse was operated separately to ensure similar exposure to isoflurane (35.7 ± 2.3 min, mean ± SD)^45^. In order to keep body temperature stable at 37 °C and monitor it during surgery, a temperature controller with heating pad (TCAT-2, Physitemp Instruments) was used. Rectal body temperature was continuously monitored during surgery using a sensor-based thermistor (36.9 ± 0.4 °C at beginning of the surgery, 37.0 ± 0.4 °C after uninephrectomy, 37.1 ± 0.3 °C five minutes after clamping the left renal pedicle and 37.1 ± 0.1 °C at the end of surgery). After right-sided uninephrectomy, ischemia was induced by clipping the pedicles of the left kidney for 20 minutes with non-traumatic aneurysm clips (FE690K, Aesculap). Reperfusion was confirmed visually. After surgery, mice had free access to water and chow. We applied body-warm sterile physiological saline solutions and preemptive analgesia with tramadol (1 mg/kg) for every mouse. Sham operation was performed in a similar manner, except for clamping the renal pedicle. Mice with bleeding during surgery, with incomplete renal reperfusion, with excessive exposure of isoflurane of any reason, with significant temperature fluctuation during surgery, or with signs for infection 24 h after IRI, were immediately euthanized and were not used for further analysis. After 24 h of reperfusion, mice were sacrificed, and kidney and blood samples were collected for further analysis. The kidneys were divided into three portions. One third of the kidney was placed in optimum cutting temperature (OCT) compound for immunohistochemistry, one third was immersed in 4% phosphate-buffered saline (PBS)-buffered formalin for histology, and the rest was snap-frozen in liquid nitrogen for RNA preparation.

### Quantitative Real-Time (qRT)-PCR

Total RNA from snap-frozen kidneys were isolated using RNeasy RNA isolation kit (Qiagen) according to manufacturer’s instruction after homogenization with a Precellys 24 homogenizator (Peqlab). RNA concentration and quality was determined by NanoDrop-1000 spectrophotometer (Thermo Fisher Scientific). Two micrograms of RNA were transcribed to cDNA (Applied Biosystems). Quantitative analysis of target mRNA expression was performed with qRT-PCR using the relative standard curve method. TaqMan and SYBR green analysis was conducted using an Applied Biosystems 7500 Sequence Detector (Applied Biosystems). The expression levels were normalized to 18S or to beta-actin. Primer sequences are provided in Supplementary Table 1.

### Western Blot

Sham and IRI-damaged kidneys were lysed with RIPA buffer (Sigma) supplemented with Complete® protease inhibitor (Roche), 1 mM phenylmethylsulfonyl fluoride (PMSF), phosphatase inhibitor cocktail 3 (Sigma) and were homogenized using a Precellys 24 homogenizator. Fifty micrograms of protein samples were separated by 12% SDS-PAGE. After wet transfer, non-specific binding sites of the nitrocellulose membrane were blocked with 5% non-fat skim milk in Tris-buffered saline containing 0.1% Tween (TBST). The membrane was then incubated with primary antibody (anti-CTH, 1:500 (ab80643) Abcam or anti-CTH carboxyl terminus rabbit polyclonal antibody that recognizes amino acids 194–398 of a rat 398-amino acid CTH protein, 1:1,000^46^ and anti-beta-actin, 1:2,000 (4970) Cell Signaling). Secondary antibody was from LI-COR Biosciences (anti-rabbit, 1:5,000). Images were acquired by Odyssey infrared imaging system (LI-COR Biosciences). Beta-actin was used as a loading control. Membranes were first probed with anti-CTH antibody and detected for their signals, and then stripped for re-probing with anti-beta-actin antibody (as loading controls). Successive stripping was confirmed by the absence of signals in the stripped membranes.

### TNF-alpha Measurement

TNF-alpha levels in the supernatants of macrophages (that were used for qRT-PCR analyses) were measured using the Mouse TNF alpha ELISA Ready-SET-Go!® Kit (eBioscience).

### H_2_S Measurement

To detect H_2_S production in the kidneys, *Cth*^+/+^ and *Cth*^−/−^ mice were euthanized and freshly isolated kidneys were incubated in PBS containing 50 μM of a recently developed fluorescent probe (Washington State Probe-1 [WSP-1], Cayman Chemical) for H_2_S^47^. After 45 min of incubation the samples were snap-frozen. Thawed samples were homogenized and centrifuged, and the supernatants were analyzed for fluorescence signals using Ex. 465 nm/Em. 525 nm using a spectrofluorometer^48^. Full spectrum was also analyzed to ensure that the measured fluorescence is indeed the product of the reaction between the probe and H_2_S. Further experiments with spiking the samples with H_2_S donor NaHS (10 and 50 μM) were performed to determine the accuracy of our measurements.

### Serum Creatinine

Blood samples were taken from left ventricle at the time of termination. After clotting on room temperature for at least 15 min blood was centrifuged at 2,000 × g for 10 min to obtain serum. Serum creatinine was measured by external clinical laboratory (Labor 28 GmbH, Berlin).

### Histology

Formalin-fixed, paraffin-embedded sections (2 μm) of kidneys were subjected to Masson’s trichrome stain using standard protocols. The severity of tubular injury was assessed by a renal pathologist who is blinded to the genotype of the mice. Tubular necrosis was evaluated in a semi-quantitative manner by determining the percentage of tubules in the cortex where epithelial necrosis, loss of the brush border, cast formation, and tubular dilation was observed. A five-point scale was used: 1, normal kidney; 2: 1 to 25%; 3: 25 to 50%; 4: 50 to 75%; and 5, 75 to 100% tubular necrosis.

### Immunofluorescence

Five-μm thick cryosections of IRI-injured kidneys were post-fixed in ice-cold acetone, air-dried, rehydrated and blocked with 10% normal donkey serum (Jackson ImmunoResearch) for 30 min. Then sections were incubated in a humid chamber overnight at 4 °C with rat anti-Ly6B.2 (Gr1) (1:300; MCA771G; AbD Serotec). The bound anti-Ly6B.2 antibody were visualized using Cy3-conjugated secondary antibody (1:500; Jackson ImmunoResearch) by incubating the sections for 1 h in a humid chamber at room temperature. Positive cells were counted in the outer medulla on five non-overlapping view fields at 200 × magnification and mean cell numbers were taken for analysis.

### Flow cytometry

To assess granulocyte and macrophage infiltration in sham-operated in IRI-injured kidneys, single cell suspension was created with GentleMacs C-tubes (Miltenyi Biotec) in the presence of 10 mg/mL collagenase IV (Sigma) and 200 U/mL DNase I (Roche) dissolved in Hank’s balanced salt solution. Dead cells were excluded from the analysis using Fixable Viability Dye eFluor 660 (eBioscience). Granulocytes and macrophages were stained with PE-conjugated anti-Ly6G (clone: 1A8, Beckton Dickinson) and eFluor450-conjugated anti-F4/80 (clone: BM8, eBioscience) antibodies, respectively. Samples were analyzed on FACSCanto II flow cytometer (Becton Dickinson). Data analysis was conducted by FlowJo (TreeStar) software.

### Preparation and activation of BM-derived macrophages

Cells were isolated from the femur and tibia of freshly euthanized *Cth*^+/+^ and *Cth*^−/−^ mice, by flushing with approximately 10 ml of activation media (RPMI1640 containing l-glutamine (Gibco), 10% (v/v) fetal calf serum (FCS), 10 mM HEPES, 50 μM beta-mercaptoethanol, 1% (v/v) penicillin/streptomycin (P/S), without colony stimulating factor (CSF)-1). Cells were then pelleted, and resuspended in monocyte differentiation media (DMEM (Gibco), 10% (v/v) FCS, 5% (v/v) adult horse serum (Cell Concepts), 1:100 non-essential amino acids (Sigma), 50 μM beta-mercaptoethanol (Sigma), with 20% (v/v) L929 conditioned media containing CSF-1 Gibco® RPMI. Conditioned media containing CSF-1 was generated by collecting the media from L929 cells (ATCC) cultured for 14 days in DMEM containing 10% (v/v) FCS, 1:100 non-essential amino acids, 10 mM HEPES and 1% (v/v) P/S. For macrophage differentiation, 10^7^ bone-marrow cells were cultivated in 50 ml of differentiation media for 7 days in sealed, hydrophobic Teflon® bags (FT FEP 100 C (DuPont), American Durafilm) at 37 °C and 10% CO_2_. The yield of BM-derived M(−) macrophages (also known as M0) from one bag was consistently approximately 7–10 × 10^7^cells with a purity of >95% (determined as F4/80+ CD11b+ cells by flow cytometry). For activation of M(−) into M(LPS+IFN-gamma) (also known as M1), BM-derived M(−) were harvested from Teflon bags, pelleted and resuspended into activation media containing LPS (100 ng/ml) and recombinant mouse IFN-gamma (20 ng/ml). For qRT-PCR analysis, 2 × 10^6^ BMD-derived M(−) and BM-derived M(LPS+IFNgamma) were plated per well of 6-well plates. In all cases, the M(−) macrophages were first allowed to rest and adhere for 2 h. Then cells were activated for 24 h at 37 °C and 5% CO_2_.

### Statistics

Statistical analysis was performed using GraphPad 5.04 (GraphPad Software) and SPSS 13.0 (SPSS) softwares. Normality of the data was evaluated by Kolmogorov-Smirnov test. To test the presence of an outlier, Grubbs’ test was used. Study groups were analyzed by one-way ANOVA using Tukey’s post-hoc test or by Games-Howell post-hoc test if homogeneity of variances was violated, with the exception of tubular necrosis data. Those were analyzed using Kruskal-Wallis test and Mann Whitney U-test. Data are presented as mean ± SEM, or median and interquartile range in case of tubular necrosis data. P values  < 0.05 were considered as statistically significant.

## Additional Information

**How to cite this article**: Markó, L. *et al.* Role of Cystathionine Gamma-Lyase in Immediate Renal Impairment and Inflammatory Response in Acute Ischemic Kidney Injury. *Sci. Rep.* **6**, 27517; doi: 10.1038/srep27517 (2016).

## Supplementary Material

Supplementary Information
