# Supplementary material for: Role of Cystathionine Gamma-Lyase in Immediate Renal Impairment and Inflammatory Response in Acute Ischemic Kidney Injury
Source: Sci Rep. 2016 Jun 8;6:27517. doi: 10.1038/srep27517 (PMC4897642; doi:10.1038/srep27517)
Supplement: Supplementary Information [file srep27517-s1.doc]

**Supplementary Information**

**Role of Cystathionine Gamma-Lyase in Immediate Renal Impairment and Inflammatory Response in Acute Ischemic Kidney Injury**

Lajos Markó^1,2^ MD, PhD, István Szijártó^1^ MD, Milos R. Filipovic^3^ PhD, Mario Kaßmann^1^ PhD, András Balogh^1,2^ MD, PhD, Joon-Keun Park^4^ PhD, Lukasz Przybyl^1^ MSc, Gabriele N’diaye^1^, Stephanie Krämer^5^ PhD, Juliane Anders^1^, Isao Ishii^6^ PhD, Dominik N. Müller^1,2,8^ PhD, Maik Gollasch^1,7,8^ MD, PhD

^1^Experimental and Clinical Research Center, a joint cooperation between the Charité Medical Faculty and the Max-Delbrück Center (MDC) for Molecular Medicine, Berlin, Germany

^2^Max-Delbrück Center (MDC) for Molecular Medicine, Berlin, Germany

^3^Friedrich-Alexander-University of Erlangen-Nürnberg, Department of Chemistry and Pharmacy, Erlangen, Germany

^4^Hannover Medical School, Hannover, Germany

^5^German Institute of Human Nutrition, Potsdam-Rehbrücke, Germany

^6^Department of Biochemistry, Graduate School of Pharmaceutical Sciences, Keio University, Tokyo, Japan

^7^Charité Campus Virchow, Nephrology/Intensive Care, Berlin, Germany;

^8^equal contribution as senior authors

The authors have declared that no conflict of interest exists.

**Corresponding Authors:**

Dr. Lajos Markó, Experimental and Clinical Research Center, Lindenberger Weg 80, 13125 Berlin, Germany, Tel: +49 30-450-540-558, Fax: +49 30-450-540-944, E-mail: lajosmarko@yahoo.com

Dr. Maik Gollasch, Experimental and Clinical Research Center, Lindenberger Weg 80, 13125 Berlin, Germany, Tel: +49 30-450-540-177, Fax: +49 30-450-553-916, E-mail: maik.gollasch@charite.de


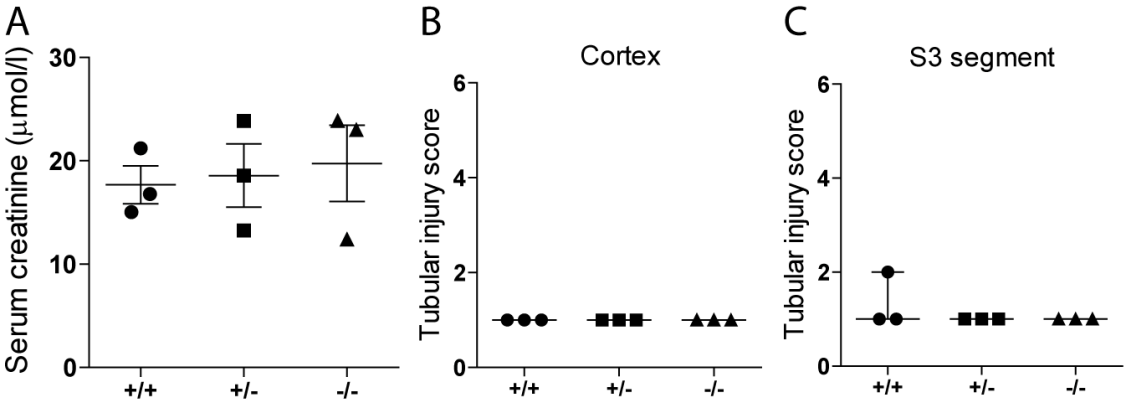


**Supplementary Figure 1.** (A) Serum creatinine levels in *Cth*^+/+^, *Cth^+^*^/-^ and *Cth^-^*^/-^ mice 24 hours after sham surgery (*n*=3 in each group). (B) Semi-quantification of cortical and (C) S3 segment tubular injury in mice underwent sham surgery.


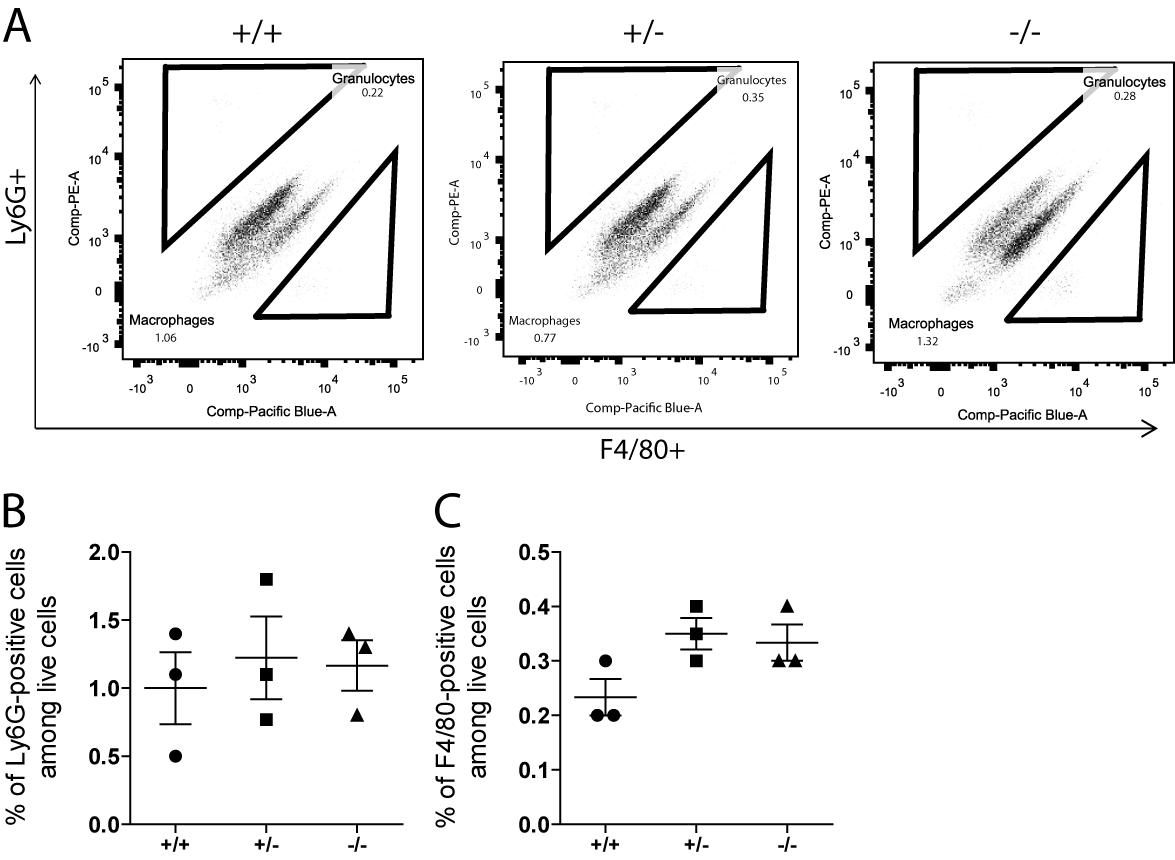


**Supplementary Figure 2. Flow cytometric detection of immune cells in sham-operated mice.** (A) Representative flow cytometry data of Ly6G-positive cells (granulocytes) and F4/80-positive cells (macrophages) in kidneys of *Cth*^+/+^, *Cth*^+/-^ and *Cth*^-/-^ mice 24 hours after sham surgery. Quantification of infiltrating (B) Ly6G-positive cells and (C) F4/80-positive cells. Values plotted are mean ± SEM (*n*=3 in each group).


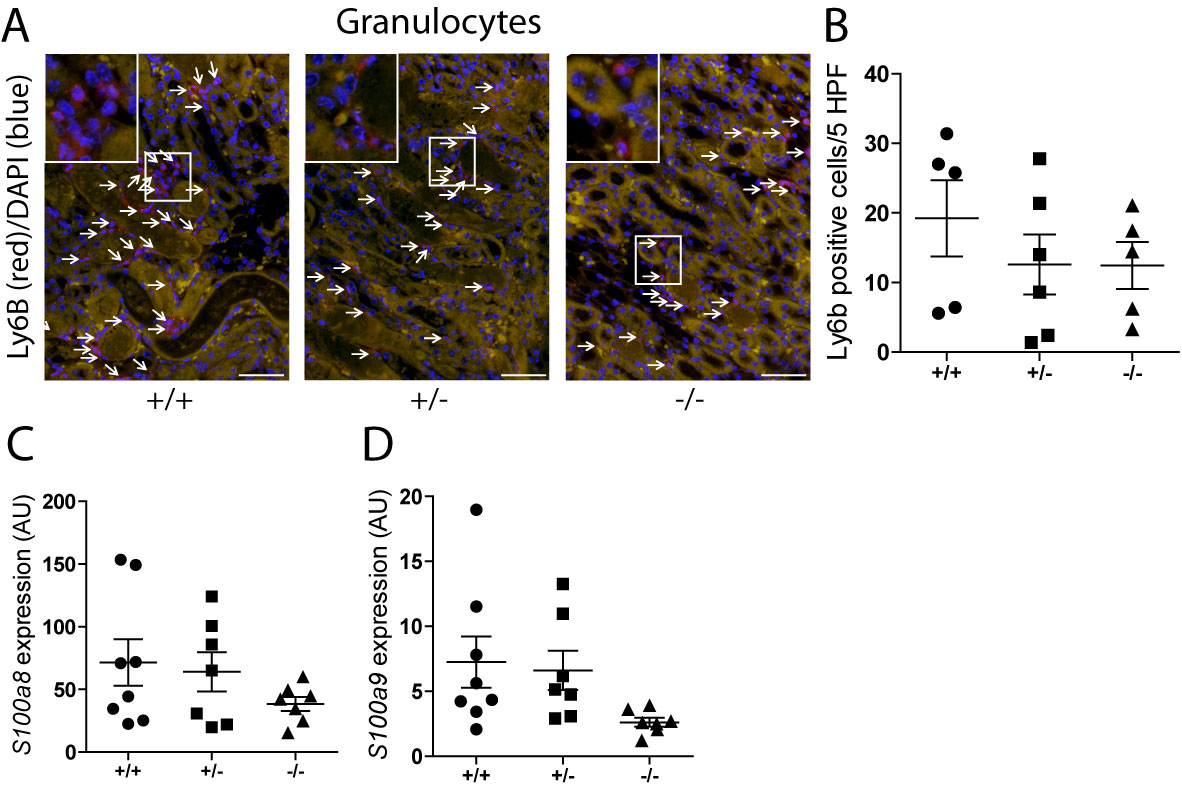


**Supplementary Figure 3. Immunofluorescent detection of Ly6B-positive cell infiltration.** (A) Immunofluorescent detection of Ly6B-positive cells (granulocytes) in ischemia/reperfusion (I/R)-injured kidneys of *Cth*^+/+^, *Cth*^+/-^ and *Cth*^-/-^ mice (×200). (B) Quantification of Ly6B-positive cells. Values plotted are mean ± SEM (n=5 in each group). Gene expression levels of (C) *S100a8* and (D) *S100a9* in I/R-injured kidneys of *Cth*^+/+^, *Cth*^+/-^ and *Cth*^-/-^ mice. Values plotted are mean ± SEM (*n*=8 in *Cth*^+/+^, *n*=7 in *Cth*^+/-^ and *n*=8 in *Cth*^-/-^ group). P=0.28 and P=0.09 for main ANOVA for *S100a8* and *S100a9*, respectively. AU, arbitrary units. HPF, high-power field.


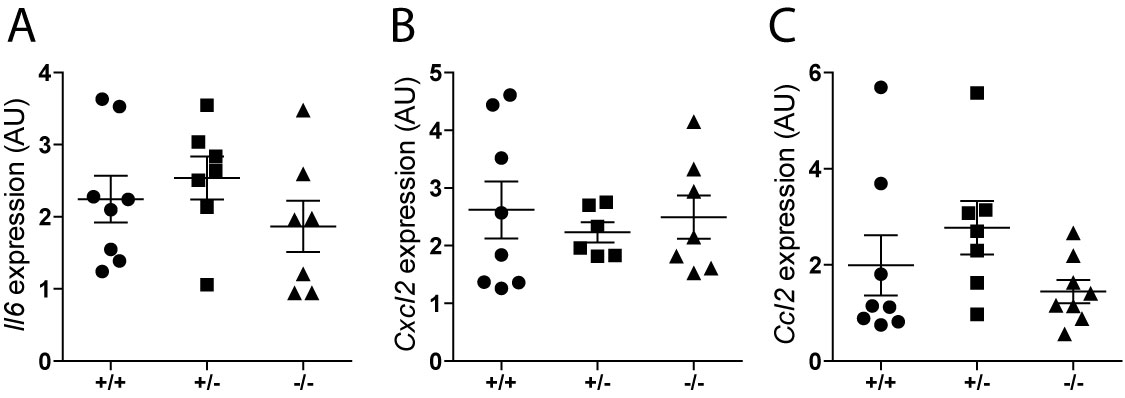


**Supplementary Figure 4.** **Renal gene expression of cytokines and chemokines.** Gene expression levels of (A) interleukin(*Il)6,* (B) chemokine (C-X-C motif) ligand (*Cxcl*)*2* and (C) chemokine (C-C motif) ligand (*Ccl*)*2* in ischemia/reperfusion-injured kidneys of *Cth*^+/+^, *Cth*^+/-^ and *Cth*^-/-^ mice. Values plotted are mean ± SEM (*n*=8 in *Cth*^+/+^, *n*=7 in *Cth*^+/-^ and *n*=8 *Cth*^-/-^ group). AU, arbitrary units.

**Supplementary Table 1.** Primer sequences used in quantitative real-time PCR.

| Gene | Forward | Probe | Reverse |
| --- | --- | --- | --- |
| 18s | 5'-ACATCCAAGGAAGGCAGCAG-3' | 5'-FAM-CGCGCAAATTACCCACTCCCGAC-TAMRA-3' | 5'-TTTTCGTCACTACCTCCCCG-3' |
| Cbs | 5'-GCAGCGCTGTGTGGTCATC-3' | - | 5'-CATCCATTTGTCACTCAGGAACTT-3' |
| Ccl2 | 5'-GGCTCAGCCAGATGCAGTTAA-3' | 5'-FAM-CCCCACTCACCTGCTGCTACTCATTCA-TAMRA-3' | 5'-CCTACTCATTGGGATCATCTTGCT-3' |
| Cth | 5'-GGCTTCCTGCCTAGTTTCCA-3' | 5'-FAM-CATTTCGCCACTCAGGCCATCCA-TAMRA-3' | 5'-TCCATTGCTCAGGCTCTTGTC-3' |
| Cxcl2 | 5'-GGGCGGTCAAAAAGTTTGC-3' | 5'-FAM-TTGACCCTGAAGCCCCCCTGGT-TAMRA-3' | 5'-TGTTCAGTATCTTTTGGATGATTTTCTG-3' |
| Havcr1 | 5'-CTGGAGTAATCACACTGAAGCAATC-3' | 5'-FAM-CTCCAGGGAAGCCGCAGAAAAACC-TAMRA-3' | 5'-GATGCCAACATAGAAGCCCTTAGT-3' |
| Il1b | 5'-AGAAGAGCCCATCCTCTGTGACTCATGG-3' | 5'-FAM-AGAAGAGCCCATCCTCTGTGACTCATGG-TAMRA-3' | 5'-CACACACCAGCAGGTTATCATCA-3' |
| Icam1 | 5'- CAGTCCGCTGTGCTTTGAGA-3' | 5'-FAM- CTGTGGCACCGTGCAGTCGTCC-TAMRA-3' | 5'- CGGAAACGAATACACGGTGAT -3' |
| Il6 | 5'- TGTCTCGAGCCCACCAGG-3' | 5'-FAM-CGAAAGTCAACTCCATCTGCCCTTCAGG-TAMRA-3' | 5'- TGCGGAGAGAAACTTCATAGCTG-3' |
| Lcn2 | 5'-TGATCCCTGCCCCATCTCT-3' | 5'-FAM-TCACTGTCCCCCTGCAGCCAGA-TAMRA-3' | 5'-GGAACTGATCGCTCCGGAA-3' |
| Mpst | 5'-CGCAGCTGGCCGTTTC-3' | - | 5'-TGTTTACTGAGCCAGGGATGTG-3' |
| S100a8 | 5'-TCACCATGCCCTCTACAAGA-3' | - | 5'-CCAATTCTCTGAACAAGTTTTCG-3' |
| S100a9 | 5'-TCAGACAAATGGTGGAAGCA-3' | - | 5'-GTCCAGGTCCTCCATGATGT-3' |
| Tnf | 5'-GGTCCCCAAAGGGATGAGAA-3' | 5'-FAM-TTCCCAAATGGCCTCCCTCTCATCA-TAMRA-3' | 5'-TGAGGGTCTGGGCCATAGAA-3' |
| Vcam1 | 5'- CTACAAGTCTACATCTCTCCCAGGAA-3' | 5’-FAM-ACAACGATCTCTGTACATCCCTCCACAAGG-TAMRA-3’ | 5'-CACAGCACCACCCTCTTGAA-3' |
